# Water footprints and crop water use of 175 individual crops for 1990–2019 simulated with a global crop model

**DOI:** 10.1038/s41597-024-03051-3

**Published:** 2024-02-14

**Authors:** Oleksandr Mialyk, Joep F. Schyns, Martijn J. Booij, Han Su, Rick J. Hogeboom, Markus Berger

**Affiliations:** Multidisciplinary Water Management group, Faculty of Engineering Technology, University of Twente, Enschede, The Netherlands

**Keywords:** Hydrology, Environmental impact, Hydrology

## Abstract

The water footprint of a crop (WF) is a common metric for assessing agricultural water consumption and productivity. To provide an update and methodological enhancement of existing WF datasets, we apply a global process-based crop model to quantify consumptive WFs of 175 individual crops at a 5 arcminute resolution over the 1990–2019 period. This model simulates the daily crop growth and vertical water balance considering local environmental conditions, crop characteristics, and farm management. We partition WFs into green (water from precipitation) and blue (from irrigation or capillary rise), and differentiate between rainfed and irrigated production systems. The outputs include gridded datasets and national averages for unit water footprints (expressed in m^3^ t^−1^ yr^−1^), water footprints of production (m^3^ yr^−1^), and crop water use (mm yr^−1^). We compare our estimates to other global studies covering different historical periods and methodological approaches. Provided outputs can offer insights into spatial and temporal patterns of agricultural water consumption and serve as inputs for further virtual water trade studies, life cycle and water footprint assessments.

## Background & Summary

The water footprint of growing a crop (further referred to as WF)—the volume of water consumed per unit of a harvested crop—is a common metric for evaluating agricultural freshwater appropriation^1^. The consumptive WF includes appropriated green water from precipitation and blue water from irrigation or capillary rise^2^. Both green and blue WFs can be used to evaluate water productivity and pressure on freshwater resources, which are key pillars of sustainable water management^3^.

Global WF patterns were first studied by Mekonnen and Hoekstra^4^ who covered around 150 individual crops focusing on the year 2000 (this dataset is further referred to as M&H2000). The authors concluded that global crop production consumes around 5.8 trillion m^3^ of green and 0.9 trillion m^3^ of blue water, collectively accounting for 87% of humanity’s water consumption^5^. To estimate WFs, they applied a soil water balance model and crop coefficient approach to obtain crop water use (CWU), defined as the volume of evapotranspired water over the growing season, and a corresponding crop yield. Both variables were calculated for rainfed and irrigated production systems separately. For rainfed areas, they estimated only green CWU since blue CWU from capillary rise was not considered. For irrigated areas, the authors estimated both green and blue CWU by performing two runs: the first run without irrigation to estimate green CWU and the second one with fully satisfied irrigation requirements to estimate the total CWU. The difference between the two was defined as blue CWU. Crop yields for both production systems were calculated based on a strong relationship between the yields, crop coefficients, and evapotranspiration^6^. Such methodology pioneered spatially explicit analysis of crop WFs, however, it also contained many limitations and uncertainties^7,8^. For instance, one study suggests that WF differences of ±30% in some regions can be expected due to uncertainty in input data^9^. Nonetheless, M&H2000 have been widely used in further studies, ranging from local evaluations of water use efficiency of specific crops to global assessments of virtual water trade^1,10^.

In the following years, several other studies simulated WFs using more advanced methods but for a small number of crops and limited spatial coverage^11–15^. More recently, Tamea *et al*.^16^ projected M&H2000 over the 1961–2016 period. Assuming CWU values remain constant in time, the authors scaled national crop yields to historical statistics allowing them to produce the desired WF time series. However, this approach not only propagates uncertainties embedded in M&H2000 but further adds more uncertainty by disregarding historical changes in CWU, which are affected by climatic variability and changes in rainfed and irrigated croplands. To address these shortcomings, Mialyk *et al*.^17^ presented a process-based global gridded crop model ACEA able to simulate the WF time series of individual crops at a high spatial resolution. This model is based on AquaCrop-OSPy—the Python version of FAO’s AquaCrop^18^—and can simulate the daily crop growth and vertical soil water balance considering local environmental conditions, crop characteristics, and farm management. Also, ACEA has integrated partitioning of green and blue water fluxes in the soil and, hence, can distinguish three consumptive WF components: green, blue from irrigation, and blue from capillary rise. Moreover, it considers historical agricultural developments by scaling rainfed and irrigated harvested areas and simulated crop production to census data. The model showed high computational efficiency when the authors simulated maize production over 1986–2016. The produced maize WFs were smaller than in M&H2000^17^ but aligned well with the broader literature, suggesting that ACEA can be further applied to provide up-to-date WF time series of other widely-grown crops.

**Fig. 1:**
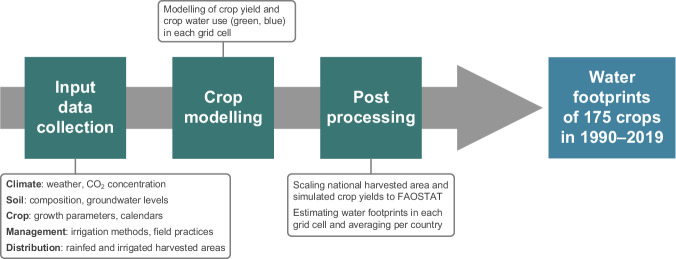
The workflow of crop water footprint simulations in this study.

Here, we simulate annual green and blue WFs of 175 individual crops over the 1990–2019 period at a 5 arcminute resolution (~8.3 km around the equator). Following the methods of Mialyk *et al*.^17^, we supply ACEA with state-of-the-art input data to calculate CWU (in mm yr^−1^) and crop yields (in t ha^−1^ yr^−1^) in each grid cell (see Fig. 1). We consider historical dynamics in the distribution of rainfed and irrigated harvested areas by combining data from SPAM2010^19^, historical datasets on cropland extent^20,21^, and national statistics from FAOSTAT^22^. The latter is also used to scale the simulated crop yields. Resulting WFs are provided in terms of consumptive unit WF (further referred to as uWF; in m^3^ t^−1^ yr^−1^) and WF of crop production (further referred to as pWF; in m^3^ yr^−1^).

We validate our data records by comparing estimates of crop yield, CWU, and WFs to other studies. Crop yields we compare with the gridded dataset by Iizumi and Sakai^23^ which covers maize, rice, wheat, and soya beans in the 1990–2014 period. Our CWU estimates we compare with: (i) Chiarelli *et al*.^24^ who provide gridded CWU of multiple crops in 2016, (ii) Jägermeyr *et al*.^25^ who also provide gridded data but for maize, rice, wheat, and soya beans in 1990–2015 simulated by multiple global crop models, and (iii) locally observed values across several crops and locations from the literature. The WF comparisons are first performed for pWF around 2000 against four global studies^4,24,26,27^ and then for uWFs of 145 crops against the mentioned earlier M&H2000.

Our datasets offer uWF, pWF, and CWU estimates at country (CSV format) and grid levels (NetCDF format) to be used for various applications including agricultural water management, environmental economics, Water Footprint and Life Cycle assessments^10^. Gridded uWF and CWU data are provided for 43 main crops that together account for 90% of global crop production. Before using our data, we advise users to familiarise themselves with interpretation guidelines and underlying uncertainties.

## Methods

### Crop model description

**Fig. 2:**
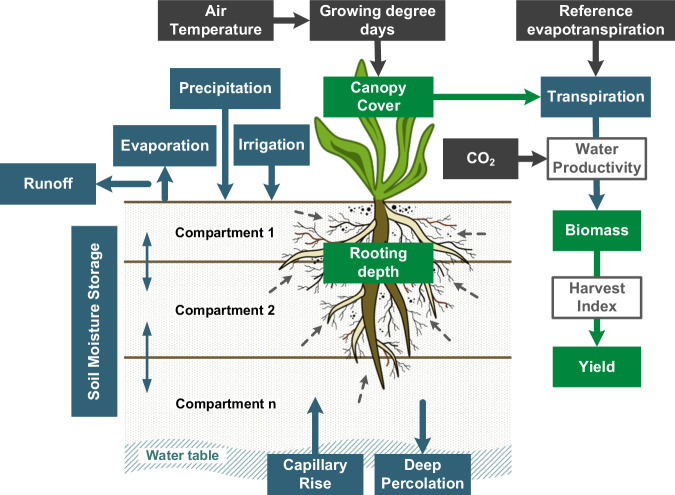
AquaCrop-OSPy simulation scheme. Green boxes indicate crop growth, blue boxes water cycle, and grey boxes climatic inputs. Adopted from Mialyk *et al*.^17^.

ACEA is based on AquaCrop-OSPy v6.1^18^ which simulates daily crop growth and the vertical soil water balance using crop, soil, climate, field and irrigation management data (see Fig. 2). Crop growth is expressed by dynamic rooting depth and canopy cover, both controlled by heat units (growing degree days). Through canopy cover, the crop transpires water abstracted by roots which drives the above-ground biomass growth via a CO_2_-adjusted water productivity parameter. Throughout the growing season, crop development is subjected to thermal and water stresses, which may slow down crop development or even lead to crop failure. Nutrient cycles, soil fertility and salinity stresses are not considered in this AquaCrop version. At the end of each growing season, the accumulated biomass is converted into a dry crop yield (in t ha^−1^ yr^−1^) via a stress-adjusted harvest index. The original soil water balance was upgraded by Mialyk *et al*.^17^ to consider green and blue water inflows through precipitation, irrigation, and capillary rise and outflows through runoff, soil evaporation, transpiration, and deep percolation. When green and blue water enters or moves through the soil profile, it mixes with prestored water at a particular depth. These fluxes are traced daily allowing for precise estimation of green and blue water volumes consumed for transpiration and soil evaporation^28^. At the end of each growing season, evapotranspired water is summed up to estimate green and blue CWU (in mm yr^−1^). For more information on AquaCrop mechanics, please refer to the original model documentation^29–31^.

AquaCrop was originally developed to simulate annual herbaceous crops. However, the model was recently applied to simulate grapes—a perennial deciduous crop^32^. The planting date was replaced with a bud break date (appearance of first green leaves), rooting depth was kept constant, and a minimum canopy cover was maintained during the leafless period to mimic shadow effects caused by branches and trunk. For our study, we replicated the same methodology for all deciduous crops. For the evergreen ones, we kept the canopy cover relatively static throughout the year. Green and blue CWU of perennial crops were estimated over the entire calendar year^2^.

### Simulation setup

We selected all 175 crops listed in FAOSTAT^33^ representing 13 crop groups: cereals, fibres, fodder crops, fruits, nuts, oil crops, others, pulses, roots, spices, stimulants, sugar crops, and vegetables. Out of those, we selected 55 core crops with sufficient input data for crop modelling, such as harvested area distribution, crop parametrisation, and calendars. The remaining 120 crops are derived from core crops based on agronomical similarities, namely genetic closeness and cropping patterns (see Table S1).

For each core crop, we run ACEA to obtain CWU and crop yields. A detailed description of the model and its input data are provided in sections “Crop model description” and “Input data”, respectively. The crop modelling was performed at a 30 arcminute resolution (~50 km around the equator) and daily timestep starting from the 1^st^ of January 1988. The earlier start allowed for a two-year warm-up period needed to generate initial soil moisture in 1990. We continued simulations until the end of 2019 including fallow periods to account for soil moisture changes in between crop-growing seasons. We then allocated 30 arcminute outputs among corresponding 5 arcminute grid cells (~8.3 km around the equator) according to the distribution of rainfed and irrigated areas from SPAM2010. All subsequent analyses, including the scaling of crop yields and WF calculation, were conducted at the latter resolution and described in “Post-processing”.

For derived crops, we assigned the same gridded CWU and crop yields as for the representative core crops but the further post-processing was based on the information specific to each derived crop.

### Input data

We summarise the main input data in Table 1. The first part of inputs covers data needed to run the crop model, which includes historical climate and atmospheric CO_2_ concentration, crop parameters, crop calendar, soil composition, groundwater levels, and irrigation management. The second part contains inputs needed in the post-processing, such as the distribution of crop-specific rainfed and irrigated harvested areas, historical cropland extent, and crop production statistics. More details on specific data inputs are provided below.

**Table 1:** Overview of input data for the crop modelling and post-processing.

Data input	Period	Timestep	Spatial resolution	Source
For crop modelling				
Climate variables	1990–2019	daily	30 arcminute	GSWP3-W5E5^34^
Atmospheric CO_2_ concentration	1990–2019	annual	global	NOAA^36^
Crop calendar	—	—	30 arcminute	Jägermeyr *et al*.^37^ and the crop-specific literature
Crop parameters	—	—	—	Default AquaCrop crop files, the crop-specific literature, and expert knowledge
Soil composition	—	—	30 arcminute	ISIMIP3^40^ based on Harmonized World Soil Database 1.2^78^
Groundwater levels	2004–2014 average	monthly	5 arcminute	Fan *et al*.^42^
Irrigation management	2004–2009 average	—	30 arcminute	Jägermeyr *et al*.^43^
For post-processing				
Harvested areas	2010	annual	5 arcminute	SPAM2010^19^, GAEZ + 2015^45^
Irrigated cropland	1985–2005	5-year	5 arcminute	HID^20^
Irrigated and rainfed croplands	1980–2017	10-year till 2000 then annual	5 arcminute	HYDE 3.2^21^
Crop production statistics	1990–2019	annual	national	FAOSTAT^22^

Historical climate data on daily rainfall, temperature, surface shortwave radiation, wind speed, and relative humidity were taken from the ISIMIP3 project which provides the bias-corrected GSWP3-W5E5 dataset^34^. These data were further used to calculate reference evapotranspiration according to the Penman-Monteith equation^35^. Atmospheric CO_2_ concentration^36^ was assumed to be uniformly distributed around the world.

Crop calendars we obtained from Jägermeyr *et al*.^37^ who provide planting and harvest dates for 18 annual crops, including two main growing seasons of rice and the distinction between winter and spring wheat. For the rest of annual crops, we took calendars either from agronomically similar crops or from the literature. ACEA could adjust these planting and harvest dates based on climatic variability. For instance, it allowed up to 15% extension of the growing season for annual crops to ensure crop maturation during cold years. On the contrary, in warm years, annual crops could accumulate heat units required for maturity faster and hence the harvest dates occurred earlier. Also, crop emergence was delayed up to one month if soil moisture content was insufficient which subsequently postponed the harvest. During fallow periods, we assumed the presence of cover crops like grasses and short weeds, which is a common practice to reduce soil erosion^38^. For deciduous perennials, we generated bud break and harvest dates based on the crop-specific temperature requirements found in the literature. Evergreen perennials were always harvested on the 31^st^ of December. We did not consider crop rotation and multi-cropping.

To parametrise core crops (listed in Table S1), we first obtained data for ten crops provided with AquaCrop by default and for another 45 core crops, we retrieved parameters either from the literature or generated ourselves based on expert knowledge. To account for regional differences in cultivars, we adjusted heat unit requirements for crop development stages in each grid cell^39^. Other differences in cultivars were not considered due to data limitations.

The soil profile had a 3 m depth subdivided into eight compartments ranging from 0.1 to 0.7 m in thickness^17^. For crops with shallow rooting depth, such as peas and cassava, the soil profile was instead limited to 2 m with seven compartments to reduce computational load. The gridded soil texture data were taken from the ISIMIP3^40^.

Shallow groundwater presence was only considered for rainfed crops since we assumed that farmers would not irrigate if crops could access water from capillary rise instead. The only exception was rice which is commonly grown under flooded conditions^41^. Daily groundwater levels were derived by interpolating monthly averages^42^. To avoid aeration stress, we assumed soils to be drained to 1 m depth in areas where groundwater reaches the surface^17^. Note that we do not consider the effects of groundwater pumping or interannual variability.

Common irrigation practices—surface, sprinkler, and drip—were defined for each crop^43^. The timing of irrigation events was controlled by thresholds for soil moisture depletion within the root zone. We defined these crop-specific thresholds according to water stress sensitivity^44^, ranging from 50% depletion for least sensitive crops (e.g. maize and chickpea) down to 25% for most sensitive ones (e.g. tomato and onion). Rice had no irrigation threshold to imitate flooding conditions and additional 0.3 m soil bunds were placed to prevent surface runoff. Irrigation thresholds for all crops are provided in Table S1. Irrigation volumes were only limited by the field capacity of the soil within the rooting zone. We do not consider water availability constraints and conveyance efficiency as we only focus on net irrigation requirements at the field level^2^. Thus, our irrigation estimates and corresponding blue CWU reflect potential values.

Distributions of crop growing areas were obtained from SPAM2010, which provides rainfed and irrigated areas for 42 crops and crop groups around 2010. Areas of alfalfa were taken from GAEZ + 2015^45^, which reports them as a part of fodder crops; areas of missing crops were copied from agronomically similar ones.

### Post-processing

For each crop, grid cell, and year, we estimated uWF by dividing green or blue CWU by corresponding crop yield^2^. We focused on the year of harvest and hence CWU could be summed over different calendar years. This happened for crops planted in a year other than harvested, such as winter wheat. Modelled yields were first converted from dry to fresh using crop water content fractions^6^. The fresh yields were subsequently scaled to match national statistics reported in the FAOSTAT database^22^ (scaling procedures are described below). Note that the latter does not provide statistics for fodder crops. Therefore, to scale their yields, we obtained an older version of the database^46^ which we linearly extrapolated to fill the missing years.

Both rainfed and irrigated uWFs were estimated by summing corresponding green and blue uWFs. For rainfed systems, blue uWF refers to blue water consumed from capillary rise; for irrigated systems, blue uWF refers to blue water either consumed from irrigation or from both capillary rise and irrigation in the case of rice. To estimate pWF, we multiplied uWF with the corresponding annual crop production. National values for uWFs were estimated by taking production-weighted averages and for CWU and crop yields by taking harvested area-weighted averages.

Our scaling procedures were similar to Mialyk *et al*.^17^ and included both the scaling of harvested areas and of crop yields. For the scaling of the former, we projected crop-specific rainfed and irrigated harvested areas from SPAM2010 over the 1990–2019 period using historical datasets on cropland extent^20,21^. The resulting annual harvested areas were then scaled to fit corresponding values from FAOSTAT. For the scaling of the latter, we multiplied fresh yields with scaled harvested areas to obtain the simulated production of a crop within a country, which was then scaled to match its counterpart in FAOSTAT. The resulting national scaling factor was equally applied over the whole country; for example, if the scaling factor is 0.5, then all rainfed and irrigated crop yields are halved. This procedure allowed us to account for historical agricultural developments that were not captured by ACEA, such as increases in fertiliser use, improvements in irrigation and machinery, or access to better crop varieties and pest control. The CWU scaling was not necessary as it is much less affected by agricultural developments compared to yields^17^.

## Data Records

We provide four types of datasets (available in *4TU.ResearchData* at 10.4121/7b45bcc6-686b-404d-a910-13c87156716a.v1^47^): national average uWFs for all 175 crops, global gridded pWFs for aggregated crop production, global gridded uWFs, and global gridded CWU. For the last two datasets, we only provide data for 43 crops that together add up to 90% of the global crop production in 2019. We also include an accompanying readme file with the metadata, supporting crop and country classifications.

### National unit water footprints of crops

Name: national_wf_crop_production_1990_2019.csv (1 file)

Format: CSV (comma separated)

Period: annual values for 1990–2019

Resolution: national values, country list according to FAOSTAT^33^

Content: green and blue uWFs and related variables of 175 crops. The list of variables is in Table 2. Users can estimate pWF by multiplying uWFs with the corresponding crop production. CWU can be derived by multiplying uWF with the corresponding crop yield and further dividing by 10.

**Table 2:** Overview of variables included in the dataset on the national water footprints of crop production.

	Variable	Unit	Description
1	crop_code		Crop code
2	crop_name		Crop name
3	country_code		Country code
4	country_name		Country name
5	year	yr	Year of harvest (crop can be planted and harvested in different years)
6	harvarea_ha	ha yr^−1^	National harvested area
7	irrigated_harvarea_fraction		Fraction of harvested area under irrigation
8	production_t	t yr^−1^	National crop production
9	crop_yield_t_ha	t ha^−1^ yr^−1^	Crop yield
10	wfg_m3_t	m^3^ t^−1^ yr^−1^	Green unit water footprint
11	wfb_cr_m3_t	m^3^ t^−1^ yr^−1^	Blue unit water footprint from capillary rise
12	wfb_i_m3_t	m^3^ t^−1^ yr^−1^	Blue unit water footprint from irrigation
13	wf_tot_m3_t	m^3^ t^−1^ yr^−1^	Total unit water footprint (sum of 10–12)

Country and crop naming and codes are aligned with FAOSTAT^33^.

### Global gridded water footprint of crop production

Name: wf_prod_{*wf_type*}_1990_2019.nc, where *wf_type* is one of: *irrigated_blue*, *irrigated_green*, *rainfed_blue*, *rainfed_green*, or *total* (5 files)

Format: NetCDF4

Period: annual values for 1990–2019 (30 bands)

Extent: 180°E–180°W and 90°S–90°N according to a WGS84 coordinate system

Resolution: 5 arcminutes (0.083333 decimal degrees), 4320 columns and 2160 rows

Content: aggregated green and blue pWF of all crops (in m^3^ yr^−1^) reported for rainfed and irrigated production systems and for both combined (total).

### Global gridded unit water footprints of crops

Name: wf_unit_{*crop_name*}_average_2010_2019.nc, where *crop_name* is one of 43 selected crop names (43 files)

Format: NetCDF4

Period: average values for 2010–2019

Extent: 180°E–180°W and 90°S–90°N according to WGS84 coordinate system

Resolution: 5 arcminutes (0.083333 decimal degrees), 4320 columns and 2160 rows

Content: seven layers with uWFs of a corresponding crop (in m^3^ t^−1^ yr^−1^) averaged over ten years. Average values are weighted by the production to reduce contribution from years with extreme values. Each layer named wf_unit_{*wf_type*} where *wf_type* is one of: *rainfed*, *rainfed_blue*, *rainfed_green*, *irrigated, irrigated_blue*, *irrigated_green*, or *total*. The layer *rainfed* is a sum *rainfed_green* and *rainfed_blue*, the layer *irrigated* is a sum *irrigated_green* and *irrigated_blue*, and the layer *total* is weighted by the production average of *rainfed* and *irrigated*.

### Global gridded crop water use of crops

Name: cwu_{*crop_name*}_average_2010_2019.nc, where *crop_name* is one of 43 selected crop names (43 files)

Format: NetCDF4

Period: average values for 2010–2019

Extent: 180°E–180°W and 90°S–90°N according to WGS84 coordinate system

Resolution: 5 arcminutes (0.083333 decimal degrees), 4320 columns and 2160 rows

Content: three layers with average CWU of a corresponding crop (in mm yr^−1^). Average values are weighted by the harvested area to reduce contribution from years with extreme values. Each layer is named cwu_{*cwu_type*} where *cwu_type* is one of: *rainfed*, *irrigated*, or *total*. The layer *total* is weighted by the harvested area average of *rainfed* and *irrigated*. Note that we report the average CWU of only one growing season—the CWU of crops planted several times a year (such as rice) are not summed up but averaged instead.

## Technical Validation

### Comparison of crop yields

Simulated yields of maize, rice, wheat, and soya beans are compared with the global gridded dataset by Iizumi and Sakai^23^—a hybrid of agricultural statistics and remote sensing products covering the 1990–2014 period. The data is reported at a 30 arcminute resolution without differentiating between rainfed and irrigated crops. Therefore, we derive corresponding values in ACEA by taking weighted by harvested area averages.

**Fig. 3:**
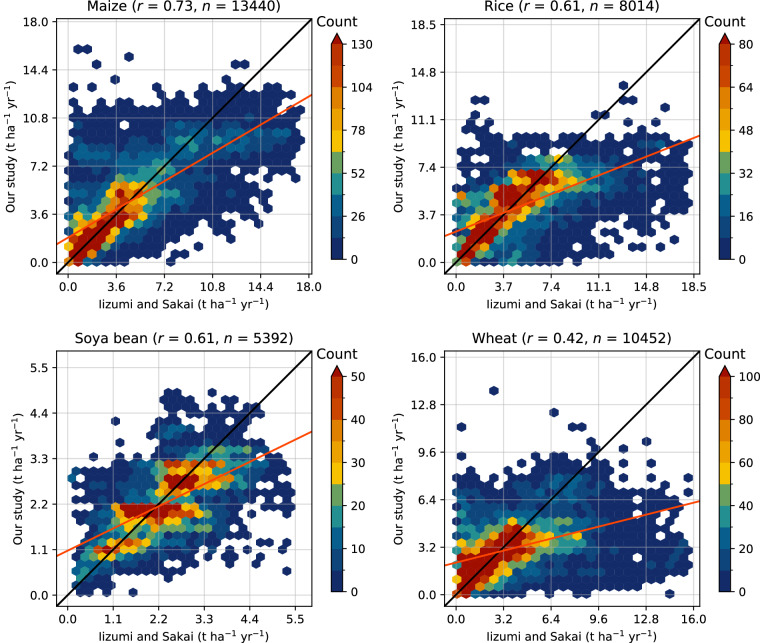
Crop yield comparisons of maize, rice, soya bean, and wheat around 2010 covering matching 30 arcminute grid cells between our study and Iizumi and Sakai^23^. Colour bars show the number of grid cells in a specific hexagon (maximum is adjusted to the sample size), *r* is the Pearson correlation coefficient, *n* is the sample size, the black line represents no difference, and the orange line is a linear regression fit. Extreme values are filtered out.

We first evaluate the agreement on historical trends. The studies agree on the global direction of crop yield changes: maize yield increased on average by 65% (69% in our study), wheat by 35% (42%), rice by 52% (56%), and soya bean by 31% (33%), which is expected as both studies are aligned with FAOSTAT. However, grid-level crop yield time series correlate only moderately—the average Pearson correlation coefficient (weighted by harvested area) ranges from 0.46 for wheat to 0.63 for maize. Next, we evaluate spatial differences between crop yield maps averaged around 2010. Global medians of grid-level differences are below 8% for all considered crops. Pearson correlation coefficients range from 0.42 for wheat to 0.73 for maize. As shown in Fig. 3, the studies demonstrate better agreement on distributions of low- to mid-range yields for maize, rice, and wheat (high concentration of red hexagons along the black line) but generally disagree on the distribution of higher yields. Overall, Iizumi and Sakai tend to report higher values as indicated by regression lines (in orange).

Moderate correlations likely stem from input data differences such as cropland extents, crop calendars, and agricultural census statistics, which was also noticed by Grogan *et al*.^45^. For instance, only 35% of ACEA’s grid cells with soya beans have corresponding values from Iizumi and Sakai. Furthermore, global crop models (including ACEA) commonly consider a limited number of non-climatic factors that affect interannual variability^48,49^. This can lead to large uncertainties in final crop yield estimates, especially in regions where such factors play a key role (e.g. socio-economic instability, natural disasters). In our study, we reduce such uncertainties with crop yield scaling (see “Post-processing”).

### Comparison of crop water use

**Fig. 4:**
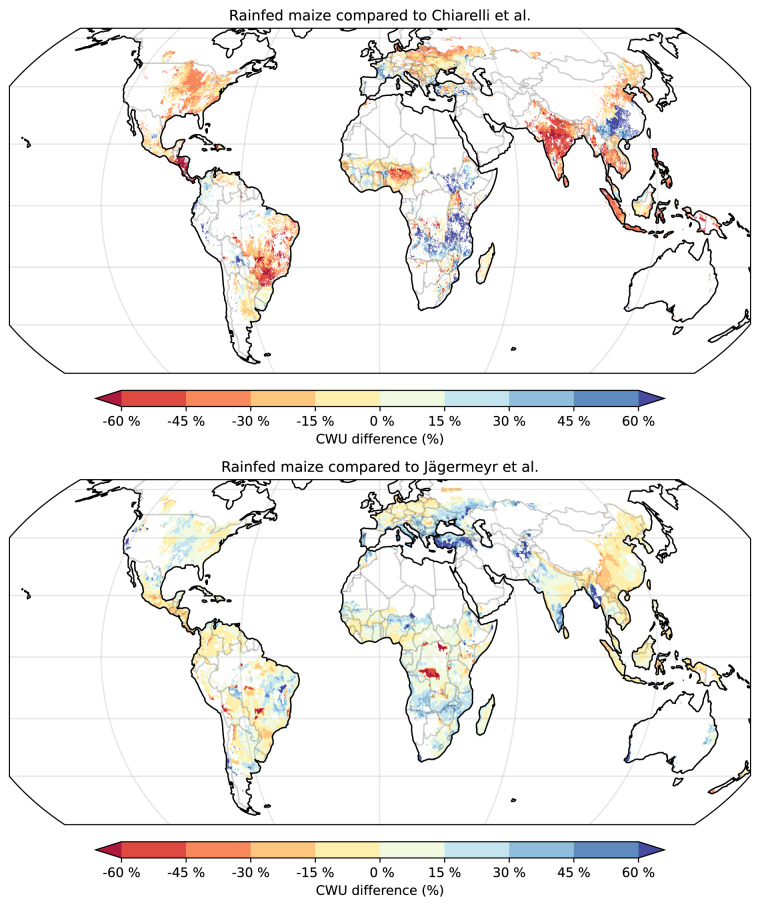
Comparison of crop water use (CWU) estimates for rainfed maize with Chiarelli *et al*.^24^ in 2016 and with Jägermeyr *et al*.^25^ averaged for 2010–2015. The latter study is represented by the mean CWU of the ensemble of four models LPJmL, EPIC-IIASA, pDSSAT, and PEPIC. Grid-level differences are calculated relative to values provided by the other studies (yellow to red colours indicate smaller values in ACEA). Extreme values are filtered out. The map is rendered using the Equal Earth projection^98^.

Similarly to crop yields, we compare our CWU estimates with other global gridded datasets. First, we compare to Chiarelli *et al*.^24^ who provide values for multiple crops in 2016 at a 5 arcminute resolution. The authors estimate CWU using the soil water balance and crop coefficients approach (described in “Background & Summary”). The maps for 13 selected crops demonstrate moderate correlations with our estimates (see Table 3). Among the rainfed crops, the two studies indicate a good agreement for sugar cane, ground nut, and potato; among the irrigated crops, the studies demonstrate high correlations for grapes, sugar cane, and soya bean. Our rainfed and irrigated CWU values are generally smaller with large regional differences between the studies, in particular for rainfed crops (see maize example in Fig. 4). The most discrepant crops are rice, wheat, and barley. They show large differences in average CWU and low spatial correlations. This is likely caused by how studies report CWU values for crops with multiple growing seasons within one calendar year. Chiarelli *et al*. may have aggregated all seasons in one value whereas we report an average value weighted by the harvested area. Other contributing factors for such discrepancies are discussed in “Comparison of crop water footprints”.

**Table 3:** Comparison of our crop water use (CWU) estimates with Chiarelli *et al*.^24^ for a set of selected crops.

Crop (with * if perennial)	Rainfed production					Irrigated production				
	Average CWU (mm)		Matching cells	Spatial correlation	Median of differences	Average CWU (mm)		Matching cells	Spatial correlation	Median of differences
	Our study	Chiarelli *et al*.				Our study	Chiarelli *et al*.			
Wheat	331	589	75.1%	0.38	−41.5%	406	778	86.5%	0.36	−46.2%
Maize	367	434	82.3%	0.36	−18.7%	461	627	89.1%	0.64	−27.1%
Rice	370	885	63.9%	0.11	−54.9%	562	935	90.3%	0.16	−35.5%
Barley	214	493	75.6%	0.09	−52.8%	325	455	85.0%	0.59	−28.1%
Sorghum	396	406	78.8%	0.55	−6.6%	549	638	80.4%	0.66	−12.2%
Soya bean	394	450	81.7%	0.50	−12.6%	471	634	88.8%	0.68	−23.9%
Potato	350	329	71.3%	0.63	+5.9%	453	568	85.1%	0.63	−20.9%
Sugar cane*	755	872	68.3%	0.66	−11.9%	1192	1291	89.7%	0.73	−6.7%
Oil palm*	908	1020	80.9%	0.60	−8.8%	1153	1532	44.2%	−0.32	−26.4%
Ground nut	412	397	75.2%	0.65	+6.0%	495	573	71.4%	0.60	−9.7%
Grapes*	353	498	42.6%	0.59	−31.5%	541	708	29.1%	0.73	−27.6%
Cotton	502	497	64.2%	0.47	−1.0%	696	842	89.9%	0.59	−15.5%
Coffee*	647	960	79.9%	0.43	−30.9%	824	1237	67.1%	0.37	−29.0%
**Average**			**72.3%**	**0.46**	−**19.9%**			**76.7%**	**0.49**	−**23.8%**

The average CWU refers to the global non-weighted average, matching cells correspond to the percentage of our grid cells which have corresponding values in the other study, spatial correlation corresponds to Pearson correlation coefficients among the matching cells, and median of differences refers to the global median of grid-level differences relative to values provided by the other study. Grid cells with values below 50 mm are excluded from this comparison.

Another study by Jägermeyr *et al*.^25^ provides gridded CWU estimates generated by several process-based gridded crop models for 1901–2016 at a 30 arcminute resolution. The simulation protocol is analogous to our study as we apply similar input data for soil, climate, and crop calendars. The authors provide irrigated crops with enough water to maintain the soil water content at field capacity, whereas in our study we use certain soil moisture depletion thresholds (see “Input data”). For our analysis, we consider the 1990–2015 period and include maize, rice (two seasons), wheat (winter and spring), and soya bean; instead of comparing to individual models, we use the mean CWU value of the ensemble of four models LPJmL, EPIC-IIASA, pDSSAT, and PEPIC. For the description of the models please refer to the study. Rainfed crops generally demonstrate high spatial correlations (see Table 4) and relatively similar CWU between the maps (see maize example in Fig. 4). Among irrigated crops, maize and soya bean are well-correlated with similar CWU, while rice and wheat show moderate correlation and larger CWU in ACEA. The latter is likely caused by differences in the way models simulate irrigation. For example, unlike other models, we account for the flooding of rice fields (see “Input data”) which likely leads to a larger CWU in our study.

**Table 4:** Comparison of crop water use (CWU) estimates for 1990–2015 between our study and Jägermeyr *et al*.^25^ for a set of selected crops.

Crop	Average CWU (mm)		Spatial correlation	Median of differences	Historical change (%)	
	Our study	Ensemble mean			Our study	Ensemble median
**Rainfed**						
Maize	363	351	0.90	+1.8%	+0.2%	−1.3%
Soya bean	379	352	0.83	+7.0%	−0.5%	−1.5%
Winter wheat	314	277	0.83	+14.2%	−2.1%	−1.1%
Spring wheat	302	250	0.88	+18.4%	−0.4%	−2.2%
Rice main season	389	368	0.89	+6.0%	+0.4%	−0.9%
Rice second	327	301	0.90	+9.5%	+1.7%	−0.3%
**Average**			**0.87**	+**9.5%**	−**0.1%**	−**1.2%**
**Irrigated**						
Maize	460	482	0.86	−8.1%	+1.1%	−1.3%
Soya bean	479	472	0.92	−0.1%	+1.9%	+0.1%
Winter wheat	432	381	0.75	+4.7%	+0.3%	−0.3%
Spring wheat	492	373	0.61	+18.7%	+1.8%	−1.7%
Rice main season	570	436	0.65	+20.7%	+0.1%	−2.0%
Rice second season	511	381	0.67	+23.8%	−0.7%	−1.8%
**Average**			**0.74**	**+10.0%**	**+0.7%**	−**1.2%**

The other study is represented by the mean CWU of the ensemble of four models LPJmL, EPIC-IIASA, pDSSAT, and PEPIC. The average CWU refers to the global non-weighted average, spatial correlation corresponds to Pearson correlation coefficients among the matching cells, median of differences refers to the global median of grid-level differences relative to values provided by the other study, and historical change reflects the average grid-level difference between values in 2011–2015 and 1990–1994. Grid cells with values below 50 mm are excluded from this comparison.

Finally, we compare the locally measured CWU of eight diverse crops from the literature to the corresponding values in ACEA. We only consider studies which report relatively similar crop calendars to ones in ACEA as CWU values are sensitive to planting and harvest dates. In total, we collected 23 values for various historical periods, production systems, and locations (see Table 5). Our estimates generally agree with other studies—the average CWU difference per crop is less than +12.1% with an overall average among 23 values of +5.0% (rainfed +1.3%, irrigated +6.3%).

**Table 5:** Comparison of crop water use (CWU) estimates between local studies and corresponding values in our study for eight crops.

	Crop (with * if irrigated)	Country	Location	Period(s)	CWU difference	Reference
1	Rice*	Philippines	14°14′ N, 121°26′ E	2008–2009	−5.3%	^79^
2	Rice*	India	29°43′ N, 76°58′ E	1994	−1.9%	^80^
3	Rice*	India	20°26′ N, 85°56′ E	2015–2016	+30.8%	^81^
4	Rice*	USA	39°21′ N, 122°05′ W	2007–2009	+9.3%	^82^
5	Rice*	China	32°21′ N, 118°68′ E	2015–2018	−12.5%	^83^
6	Wheat				+16.3%	
7	Wheat*	China	39°36′ N, 116°48′ E	2005–2008	−2.1%	^84^
8	Wheat*	India	30°56′ N, 75°52′ E	2006–2008	+9.1%	^85^
9	Maize (corn)*	USA	36°69′ N, 108°31′ W	2011–2014, 2017	+6.1%	^86^
10	Maize (corn)*	USA	41°09′ N, 96°28′ W	2001–2005	+9.1%	^87^
11	Maize (corn)				+1.3%	
12	Soya bean*				+3.5%	
13	Soya bean				−8.9%	
14	Soya bean*	USA	33°42′ N, 90°55′ W	2016	+13.3%	^88^
15	Seed cotton*	Syria	36°01′ N, 36°56′ E	2004–2006	+6.8%	^89^
16	Seed cotton*	China	85°49′ E, 44°17′ N	2010	−0.9%	^90^
17	Sorghum*	Spain	39°03′ N, 2°05′ W	2007, 2010	−3.1%	^91^
18	Sorghum	USA	36°13′ N, 97°10′ W	2011–2013	−0.6%	^92^
19	Sorghum*	USA	29°13′ N, 99°45′ W	2006–2008	+28.8%	^93^
20	Sugar cane*	Brazil	20°43′ S, 51°16 W	2016–2018	+21.0%	^94^
21	Sugar cane*	India	19°52′ N, 74°58′ E	2015–2016	+3.1%	^95^
22	Oil palm	Indonesia	4°16′ S, 105°36′ E	2017–2019	−0.4%	^96^
23	Oil palm	Malaysia	01°44′ N, 103°32′ E	2006	+0.1%	^97^

CWU difference is relative to the value provided by the other study.

### Comparison of crop water footprints

For green and blue pWFs, we provide comparisons to four global studies^4,24,26,27^ that report corresponding estimates around the year 2000 (see Table 6). ACEA’s total pWFs are consistently smaller with the blue pWF generally demonstrating larger discrepancies. Shares of green water in the total pWF are relatively similar among studies. When looking at specific crops, ACEA also shows consistently smaller total pWFs with substantial variation among the studies.

**Table 6:** Comparison of water footprints of crop production and related variables to other global studies^4,24,26,27^.

Crops	Water footprint type	This study (billion m^3^)			Mekonnen and Hoekstra	Chiarelli *et al*.	Siebert and Döll	Liu and Yang
		1990	2019	Around 2000				
Maize	Green	480	705	508	597 (+18%)	627 (+24%)	585 (+15%)	—
	Blue	35	59	45	51 (+13%)	76 (+68%)	72 (+60%)	—
	Total	515	764	553	648 (+17%)	704 (+27%)	658 (+19%)	—
Rice	Green	404	424	398	679 (+71%)	636 (+60%)	634 (+59%)	—
	Blue	232	266	261	202 (−23%)	273 (+5%)	307 (+18%)	—
	Total	636	690	659	881 (+34%)	909 (+38%)	941 (+43%)	—
Soya bean	Green	234	488	314	351 (+12%)	381 (+21%)	382 (+22%)	—
	Blue	8	12	11	12 (+7%)	14 (+25%)	17 (+54%)	—
	Total	242	500	325	363 (+12%)	395 (+22%)	399 (+23%)	—
Sugar cane	Green	127	222	140	180 (+29%)	162 (+16%)	173 (+23%)	—
	Blue	45	75	60	74 (+24%)	65 (+9%)	69 (+16%)	—
	Total	172	297	200	254 (+27%)	227 (+14%)	241 (+21%)	—
Fodder crops	Green	625	563	541	559 (+3%)	567 (+5%)	659 (+22%)	—
	Blue	63	61	59	72 (+22%)	92 (+55%)	102 (+71%)	—
	Total	688	623	601	631 (+5%)	659 (+10%)	761 (+27%)	—
Total crop production	Green	4535	5912	4712	5771 (+22%)	5414 (+15%)	5505 (+17%)	5011 (+6%)
	Blue	699	873	815	899 (+10%)	1068 (+31%)	1180 (+45%)	927 (+14%)
	Net irrigation	774	977	898	899 (0%)	1068 (+19%)	1180 (+31%)	927 (+3%)
	Total	5234	6785	5527	6670 (+21%)	6482 (+17%)	6685 (+21%)	5938 (+7%)
	Percentage of green	86.7%	87.1%	85.2%	86.5%	83.5%	82.3%	84.4%

All non-percentage numbers are provided in billion cubic metres. Values in brackets show a relative difference between our and the other studies.

Such discrepancies stem from differences in crop maps and CWU estimates (since pWF is the multiplication of the two). We use SPAM2010 crop maps adjusted to represent historical dynamics (see “Post-processing”), whereas the other studies use static MIRCA2000 maps^50^. This leads to a mismatch in the distribution and size of rainfed and irrigated areas. For instance, we estimate 11–15% smaller global irrigated harvested area around 2000, which most likely leads to smaller blue pWFs. ACEA’s estimate for 2005–2009, however, deviates by less than 7% from the values reported in the literature^20,43,51,52^. As for the CWU estimates, multiple factors can explain the differences among the studies. Below, we listed the factors that most likely lead to smaller CWU in ACEA:

- *Crop modelling*. ACEA simulates both the vertical soil water balance and crop growth (see “Crop model description”), with the latter being temperature and water-dependent and constrained by water and heat stresses. Additionally, water volumes available for evaporation and transpiration are controlled by soil characteristics and variable rooting depth. The other studies however model only the soil water balance with crop development being expressed by predefined crop coefficients and rooting depth. They also consider the limited effects of water deficit and do not account for heat stress. Consideration of such biophysical processes in ACEA likely leads to smaller CWU, especially in water-limited areas. When compared to the other process-based crop models, our estimates agree well (see “Comparison of crop water use”).
- *Irrigation management*. ACEA triggers irrigation once the soil water content within the rooting zone drops below a certain threshold which depends on the crop’s tolerance to water stress (see “Input data”). Irrigation volume is controlled by the type of irrigation system (surface, sprinkler, or drip) and the maximum holding capacity of the soil. The other studies trigger irrigation once actual evapotranspiration supported by soil moisture is below the potential one (under no water stress); irrigation volume is equal to the difference between the actual and potential evapotranspiration. This likely results in smaller irrigation volumes and hence blue CWU in ACEA (see Table 6). When compared to the global agro-hydrological models, our global net irrigation volume is also smaller. In ACEA, this estimate for 2004–2009 is 959 km^3^ compared to 1257 km^3^ in LPJmL^43^ and for 2000 it is 952 km^3^ compared to 1098 km^3^ in PCR-GLOBWB^53^. Larger estimates by both studies are likely caused by their simplified crop representation, different land use data, smaller soil moisture depletion thresholds, and consideration of additional water consumption from canopy interception and conveyance losses. The latter can be as high as 30% for open canal systems^43^.
- *Green-blue partitioning*. ACEA has green-blue partitioning integrated into the daily soil water balance calculations—a recommended method for a more precise way of estimating green and blue CWU^28^. The other studies do this partitioning by equalising green CWU to actual rainfed evapotranspiration and blue one to the difference between the latter and potential evapotranspiration under fully satisfied irrigation water requirements. This implies that all blue water is immediately consumed once irrigated. However, in reality, some water ends up being lost to runoff, stored in the soil, or drained to layers deeper than the rooting zone and thus not consumed in WF terms. Moreover, the daily fluxes of green and blue water within the soil profile affect the fractions of both water types in the final CWU. For example, some blue water volume irrigated in a given season can be stored in the soil and consumed for evapotranspiration in subsequent seasons, potentially decreasing irrigation needs. These dynamics are considered in ACEA and ultimately result in a 10% smaller blue CWU at the global level compared to the net irrigation requirement (see Table 6).
- *Initial soil moisture*. Another assumption concerns the initial soil moisture which is commonly generated by starting simulations several years in advance. However, Mekonnen and Hoekstra^4^ simulated only one average year and assumed the initial soil moisture was at field capacity holding only green water. This likely leads to an overestimation of green and an underestimation of blue CWU. The other considered studies simulated multiple years including the fallow periods which generated more reasonable soil moisture conditions. However, they did not account for daily green and blue water fluxes within the soil, which resulted in different compositions of the final CWU.

Besides the mentioned above, other factors certainly contribute to CWU differences but the relative contribution of such factors is unclear:

- *Different input datasets for climate and soil texture*. Climate variables affect many biophysical processes in ACEA, such as the water deficiency effect on canopy development or pollination failure due to extreme heat. Soil texture affects hydraulic properties and hence water balance, e.g. sandy soils need more irrigation as they store less water and drain faster compared to loamy and clayey ones. Hence, any differences in these inputs (amplified by other factors mentioned earlier) may lead to substantial CWU differences.
- *Consideration of capillary rise*. Among these studies, only ACEA considers shallow groundwater, which provides blue water for rainfed crops via capillary rise. This may lead to larger rainfed CWU in countries with the widespread presence of shallow groundwater like the Netherlands or Bangladesh. On the other hand, we also consider shallow groundwater for irrigated rice, which likely reduces its irrigation needs.
- *Crop parametrisation*. In our study, we cover 175 crops of which 55 are simulated as individual crops and the rest are derived (see Table S1). Most other studies simulate only 26 individual crops (or groups) as provided by MIRCA2000, which likely results in large uncertainties. For instance, the crop group ‘other annual crops’ in MIRCA2000 contains all vegetables which can vary greatly in crop parameters and calendars. This can lead to both different daily evapotranspiration rates and growing season periods. Moreover, we allow for adjustments in growing seasons to provide more time for maturity in cold years and less time in warm years (see “Input data”).

As a final step, we compare our uWF estimates against the values of 145 corresponding crops from M&H2000^4^. Similarly to the total pWF, ACEA simulated 20% smaller values on average. Nevertheless, the crop-by-crop correlation is high—the Pearson correlation coefficient is 0.97. Since crop yields in both studies undergo scaling to historical statistics (see “Post-processing”), we presume that most discrepancies between uWFs stem from CWU differences, as explained earlier.

## Usage Notes

### Potential applications

You can use our WF datasets for various needs. The foremost purpose is to study historical patterns in crop water productivity (uWFs) and water consumption (pWFs). The latter can be combined with water availability data to evaluate water scarcity^54,55^. Moreover, our data serves as a basis for performing Water Footprint Assessment (WFA) and Life Cycle Assessment (LCA) of crop-derived products or industrial products containing agricultural production in their supply chains^10^. For example, WF data are required in the ISO 14044:2006 standard for the environmental impact assessment of a product^56^. Furthermore, coupled with trade statistics or Multi-Regional Input-Output Tables (MRIO), our datasets enable analysis of virtual water trade^57^.

Datasets on CWU can serve you as a reference point for assessing regional crop water needs. For instance, you can estimate how much water is needed to cultivate variable areas of rainfed and irrigated crops in a specific region. Coupled with optimisation algorithms, this can facilitate the sustainable allocation of water resources.

Note that national outputs are less affected by inherent biases and uncertainties compared to gridded counterparts (see “Limitations and uncertainties”). When using the latter, we recommend aggregating data to regional levels (e.g. hydrologic or administrative units). You should also be aware that we represent historical changes in national borders, for example, the post-Soviet countries are covered from 1992 onwards.

### Interpreting data

In light of limitations and uncertainties, you should critically assess the applicability of our datasets for a given task before drawing any conclusions. To start, we only provide consumptive green and blue WFs. To analyse the total freshwater appropriation, you should also include water pollution as represented by the grey WF^4^. When assessing consumptive WFs, keep the following aspects in mind:

- *Blue water consumption is not the same as irrigation*. Blue water consumption from irrigation refers only to the potential volume of irrigated water consumed for transpiration and evaporation. Water volumes remained in the soil, returned to the system, or lost during conveyance are not included. Therefore, blue water consumption is different from irrigation demand or withdrawal. Moreover, irrigation volumes in our model are controlled by constant soil moisture thresholds and irrigation practices while not being constrained by blue water availability (see “Input data”). As a result, our irrigation estimates and hence blue WFs reflect potential rather than actual values.
- *Green water versus blue water*. Due to the different nature and utility of green and blue water, stating that one is more valuable for humankind than the other is problematic. Nonetheless, people predominantly focus on blue water resources—the primary source for domestic and industrial freshwater supply and hence a well-studied and regulated natural resource. On the contrary, green water resources are generally taken for granted and neglected by water policies^58^ despite being the main water source for crop production (see Table 6) and playing a pivotal role in ecosystem functioning, e.g. soil health and erosion control, carbon sequestration, water and nutrient recycling. Moreover, all blue water bodies (lakes, rivers, aquifers) originate from green water delivered via precipitation and runoff^59^. Therefore, changes in green water consumption may affect the water cycle and potentially lead to further adverse effects on ecosystems. In WF terms, this means that both green and blue WFs of crops should be critically assessed on a case-to-case basis^2,60^, particularly in the regions experiencing water scarcity^54,55^.
- *Comparing uWFs between crops*. We recommend selecting crops with similar nutritional and economic values. Once selected, you should convert uWF from m^3^ per tonne to units that adequately represent values of these crops^4,61^. For example, protein-rich crops can be compared in terms of m^3^ per gramme of protein or energy-dense crops in terms of m^3^ per kcal or GJ.
- *Comparing uWFs between regions*. Smaller uWF of a crop in Region A compared to Region B indicates more efficient crop production and (or) better climatic suitability. Due to the latter, you should rather compare this uWF to an appropriate benchmark level tailored to the local climate type^62,63^. This allows for assessing production efficiency limited by climatic suitability. If the value is above the corresponding benchmark (not efficient), you can evaluate the potential degree of uWF reduction. Note that this reduction can be also limited by non-environmental factors such as lack of human, economic, and institutional capacity^64^ or access to better agricultural inputs including crop varieties, machinery, fertilisers, and pest control^65^. Also, smaller uWFs may come at the expense of carbon, chemical, or biodiversity footprints^66,67^.

### Limitations and uncertainties

Uncertainties arise at each step of our study (see Fig. 1), starting with the quality of input data and ending with the post-processing of crop model outputs. Quantifying these uncertainties would require a large number of additional simulations using different input data and crop models. Such analysis would go beyond the scope of our study as we only aim to use one specific crop model and set of input data to estimate crop WFs and compare the resulting estimates to the broader literature. Thus, in this section, we do not quantify uncertainties but briefly discuss their main sources and suggest ways of reducing them in future studies.

The primary source of uncertainty originates from the quality and resolution of input data. Most inputs were obtained at 30 arcminute resolution (see “Input data”), reflecting average environmental conditions in an area of approximately 50 × 50 km. This negates spatial variability within grid cells. For instance, local variability in soil composition can substantially affect water availability and hence CWU and crop yields^68^. In areas with shallow groundwater, we consider only multi-year average monthly levels which neglects interannual dynamics, such as the effects of pumping. Crop calendars provide only approximate planting and harvest dates over large spatial scales. This introduces uncertainty in the actual start and duration of growing seasons, which likely propagates into CWU estimates. These limitations can be minimized in future studies once more accurate input data become available.

Another source of uncertainty lies in the setup and outputs of the crop model. We based ACEA on AquaCrop which was originally developed to study the site-based water productivity of crops calibrated to local agro-climatic conditions^68^. To enable global simulations, we derived a universal set of crop parameters from the literature and only calibrated crop development stages to match reported crop calendars in each grid cell (see “Input data”). This calibration did not account for differences in other crop parameters among cultivars, such as the maximum canopy cover, crop coefficients, or rooting depth—even though these are important in regions with sub-optimal agricultural conditions^69,70^. In fallow periods, we assumed the presence of cover crops like grasses and short weeds whereas some farmers may leave soils bare. We also assumed a common soil moisture-based rule to initiate irrigation application, while the farmer’s decision on timing and volume of irrigation depends on local environmental and economic conditions. Additionally, our version of AquaCrop could not explicitly simulate fertiliser inputs. Instead, we applied yield scaling to consider the combined effect of fertiliser use and other agricultural developments at the national level (see “Post-processing”). The above-mentioned uncertainties can be reduced by utilising crop yield and CWU estimates from an ensemble of crop models^71,72^, but such endeavour would make global assessments impractical due to large computational requirements. Additionally, the uncertainties can be further minimized by coupling crop models with remote sensing products^73–75^. Such an approach is still in the early development stage but could be implemented in future updates of WF datasets.

Lastly, the post-processing of outputs introduced additional uncertainty when harvested areas and crop production were scaled to national statistics from FAOSTAT (see “Post-processing”). The scaling of harvested areas added historical dynamics to otherwise static maps of rainfed and irrigated areas; the scaling of crop production allowed accounting for historical agricultural developments. Both scaling procedures included multiple assumptions affecting the reliability of the final WF estimates. For instance, there was no differentiation between production systems in FAOSTAT and, hence, both rainfed and irrigated crop yields were scaled with the same scaling factors. In future updates, these factors could be adjusted according to farm sizes^76^ and farming intensity^19^.

### Supplementary information

Supplementary information

## Data Availability

The source code for AquaCrop-OSPy v6.1—the crop model upon which ACEA is based—is freely available via github.com/aquacropos/aquacrop. The source code and most inputs for ACEA (version 2.0) are available via Zenodo^77^. Note that some input datasets are not included but can be directly obtained from the original sources instead. You can find brief instructions and references to input datasets in the readme file.
