# Supplementary material for: Water footprints and crop water use of 175 individual crops for 1990–2019 simulated with a global crop model
Source: Sci Data. 2024 Feb 14;11:206. doi: 10.1038/s41597-024-03051-3 (PMC10866886; doi:10.1038/s41597-024-03051-3)
Supplement: Supplementary file 1 — Supplementary information [file 41597_2024_3051_MOESM1_ESM.pdf]

Supplementary information

Table S1. List of considered crops. Names, groups, and codes of crops are taken from FAO's classification[1], "irrigation threshold" refers to the soil water content below which irrigation is triggered, and "method" states whether the corresponding crop is modelled or derived from other crops.

#	FAO name	FAO group	FAO code	Irrigation threshold	Method	Reference
1	Barley	Cereal	44	50%	Modelled	Default from AquaCrop, [2]
2	Cereals n.e.c.	Cereal	108	50%	Modelled	Generic annual cereal grass similar to #79
3	Maize (corn)	Cereal	56	50%	Modelled	Default from AquaCrop
4	Millet	Cereal	79	50%	Modelled	[3,4]
5	Oats	Cereal	75	50%	Modelled	[5]
6	Rice	Cereal	27	100%	Modelled	Default from AquaCrop
7	Sorghum	Cereal	83	50%	Modelled	Default from AquaCrop
8	Wheat	Cereal	15	50%	Modelled	Default from AquaCrop
9	Other fibre crops, raw, n.e.c.	Fibres	821	50%	Modelled	Generic perennial fibre crop similar to #260
10	Seed cotton, unginned	Fibres	328	50%	Modelled	Default from AquaCrop
11	Forage and silage, alfalfa	Fodder crops	641	50%	Modelled	Default from AquaCrop
12	Apples	Fruits	515	50%	Modelled	[6]
13	Avocados	Fruits	572	50%	Modelled	[7]
14	Bananas	Fruits	486	65%	Modelled	[8]
15	Grapes	Fruits	560	60%	Modelled	[9]
16	Oranges	Fruits	490	50%	Modelled	[10]
17	Other berries and fruits of the genus vaccinium n.e.c.	Fruits	558	50%	Modelled	Generic perennial temperate crop similar to #560, [11]
18	Other tropical fruits, n.e.c.	Fruits	603	50%	Modelled	Generic perennial tropical crop similar to #217, [12]
19	Plantains and cooking bananas	Fruits	489	65%	Modelled	Generic perennial tropical crop similar to #486
20	Cashew nuts, in shell	Nuts	217	50%	Modelled	[13]
21	Coconuts, in shell	Oil	249	50%	Modelled	[14,15]
22	Groundnuts, excluding shelled	Oil	242	40%	Modelled	[16]
23	Karite nuts (sheanuts)	Oil	263	50%	Modelled	Generic perennial tropical crop similar #254
24	Oil palm fruit	Oil	254	50%	Modelled	[17]
25	Olives	Oil	260	50%	Modelled	[18]
26	Other oil seeds, n.e.c.	Oil	339	50%	Modelled	Generic annual temperate crop similar to #270 and #267
27	Rape or colza seed	Oil	270	50%	Modelled	[19]
28	Sesame seed	Oil	289	40%	Modelled	[20]
29	Soya beans	Oil	236	50%	Modelled	Default from AquaCrop
30	Sunflower seed	Oil	267	50%	Modelled	Default from AquaCrop
31	Natural rubber in primary forms	Others	836	50%	Modelled	[21,22]
32	Unmanufactured tobacco	Others	826	50%	Modelled	[23]
33	Beans, dry	Pulses	176	50%	Modelled	[24]
34	Chick peas, dry	Pulses	191	50%	Modelled	[25]
35	Cow peas, dry	Pulses	195	50%	Modelled	[26,27]

36	Lentils, dry	Pulses	201	50%	Modelled	Generic annual temperate crop similar to #176
37	Other pulses n.e.c.	Pulses	211	50%	Modelled	Generic annual temperate crop similar to #201
38	Peas, dry	Pulses	187	50%	Modelled	[28]
39	Pigeon peas, dry	Pulses	197	50%	Modelled	Generic annual temperate crop similar to #187
40	Cassava, fresh	Roots	125	60%	Modelled	[29]
41	Potatoes	Roots	116	60%	Modelled	[30]
42	Sweet potatoes	Roots	122	60%	Modelled	[31]
43	Taro	Roots	136	60%	Modelled	[32]
44	Yams	Roots	137	60%	Modelled	[33]
45	Cocoa beans	Stimula nts	661	50%	Modelled	[34,35]
46	Coffee, green	Stimula nts	656	50%	Modelled	[36–38]
47	Tea leaves	Stimula nts	667	50%	Modelled	[39]
48	Sugar beet	Sugar crops	157	50%	Modelled	[40]
49	Sugar cane	Sugar crops	156	60%	Modelled	[41]
50	Chillies and peppers, green (Capsicum spp. and Pimenta spp.)	Vegeta bles	401	65%	Modelled	[42]
51	Onions and shallots, dry (excluding dehydrated)	Vegeta bles	403	75%	Modelled	[43]
52	Other vegetables, fresh n.e.c.	Vegeta bles	463	60%	Modelled	Generic fruit vegetable similar to #388 and #401
53	Tomatoes	Vegeta bles	388	60%	Modelled	Default from AquaCrop
-	Vegetables, leafy	Vegeta bles	-	60%	Modelled	Generic leafy vegetable similar to #463, [44]
-	Vegetables, tuber	Vegeta bles	-	60%	Modelled	Generic tuber vegetable similar to #463 and #157
54	Buckwheat	Cereal	89		Derived	Based on #108
55	Canary seed	Cereal	101		Derived	Based on #108
56	Fonio	Cereal	94		Derived	Based on #108
57	Mixed grain	Cereal	103		Derived	Based on #108
58	Quinoa	Cereal	92		Derived	Based on #56
59	Rye	Cereal	71		Derived	Based on #15
60	Triticale	Cereal	97		Derived	Based on #15
61	Abaca, manila hemp, raw	Fibres	809		Derived	Based on #486
62	Agave fibres, raw, n.e.c.	Fibres	800		Derived	Based on #821
63	Flax, processed but not spun	Fibres	773		Derived	Based on #339
64	Jute, raw or retted	Fibres	780		Derived	Based on #187
65	Kenaf, and other textile bast fibres, raw or retted	Fibres	782		Derived	Based on #187
66	Ramie, raw or retted	Fibres	788		Derived	Based on #187
67	Sisal, raw	Fibres	789		Derived	Based on #821
68	True hemp, raw or retted	Fibres	777		Derived	Based on #339
69	Beets for fodder	Fodder crops	647		Derived	Based on #157
70	Cabbage for fodder	Fodder crops	644		Derived	Based on leafy vegetable
71	Carrots for fodder	Fodder crops	648		Derived	Based on tuber vegetable

72	Clover for forage	Fodder crops	640		Derived	Based on #641
73	Forage and silage, green oilseeds	Fodder crops	642		Derived	Based on #339
74	Forage and silage, maize	Fodder crops	636		Derived	Based on #56
75	Forage and silage, rye grass	Fodder crops	638		Derived	Based on #641
76	Forage and silage, sorghum	Fodder crops	637		Derived	Based on #83
77	Mixed Grasses and Legumes	Fodder crops	645		Derived	Based on #44
78	Other forage products, n.e.c.	Fodder crops	651		Derived	Based on #641
79	Other grasses for forage	Fodder crops	639		Derived	Based on #641
80	Other legumes for forage	Fodder crops	643		Derived	Based on #463
81	Swedes for fodder	Fodder crops	649		Derived	Based on tuber vegetable
82	Turnips for forage	Fodder crops	646		Derived	Based on tuber vegetable
83	Vegetables and roots fodder	Fodder crops	655		Derived	Based on #463
84	Apricots	Fruits	526		Derived	Based on #515
85	Blueberries	Fruits	552		Derived	Based on #558
86	Cashewapple	Fruits	591		Derived	Based on #217
87	Cherries	Fruits	531		Derived	Based on #515
88	Cranberries	Fruits	554		Derived	Based on #558
89	Currants	Fruits	550		Derived	Based on #558
90	Dates	Fruits	577		Derived	Based on #217
91	Figs	Fruits	569		Derived	Based on #560
92	Gooseberries	Fruits	549		Derived	Based on #558
93	Kiwi fruit	Fruits	592		Derived	Based on #515
94	Lemons and limes	Fruits	497		Derived	Based on #490
95	Locust beans (carobs)	Fruits	461		Derived	Based on #490
96	Mangoes, guavas and mangosteens	Fruits	571		Derived	Based on #603
97	Other citrus fruit, n.e.c.	Fruits	512		Derived	Based on #490
98	Other fruits, n.e.c.	Fruits	619		Derived	Based on #217
99	Other pome fruits	Fruits	542		Derived	Based on #515
100	Other stone fruits	Fruits	541		Derived	Based on #515
101	Papayas	Fruits	600		Derived	Based on #603
102	Peaches and nectarines	Fruits	534		Derived	Based on #515
103	Pears	Fruits	521		Derived	Based on #515
104	Persimmons	Fruits	587		Derived	Based on #515
105	Pineapples	Fruits	574		Derived	Based on #490
106	Plums and sloes	Fruits	536		Derived	Based on #515
107	Pomelos and grapefruits	Fruits	507		Derived	Based on #490
108	Quinces	Fruits	523		Derived	Based on #515
109	Raspberries	Fruits	547		Derived	Based on #558
110	Sour cherries	Fruits	530		Derived	Based on #515
111	Strawberries	Fruits	544		Derived	Based on #558
112	Tangerines, mandarins, clementines	Fruits	495		Derived	Based on #490

113	Almonds, in shell	Nuts	221		Derived	Based on #515
114	Areca nuts	Nuts	226		Derived	Based on #249
115	Brazil nuts, in shell	Nuts	216		Derived	Based on #661
116	Chestnuts, in shell	Nuts	220		Derived	Based on #515
117	Hazelnuts, in shell	Nuts	225		Derived	Based on #515
118	Kola nuts	Nuts	224		Derived	Based on #661
119	Other nuts (excluding wild edible nuts and groundnuts), in shell, n.e.c.	Nuts	234		Derived	Based on #515
120	Pistachios, in shell	Nuts	223		Derived	Based on #217
121	Walnuts, in shell	Nuts	222		Derived	Based on #515
122	Castor oil seeds	Oil	265		Derived	Based on #339
123	Hempseed	Oil	336		Derived	Based on #339
124	Jojoba seeds	Oil	277		Derived	Based on #263
125	Kapok fruit	Oil	310		Derived	Based on #263
126	Linseed	Oil	333		Derived	Based on #339
127	Melonseed	Oil	299		Derived	Based on #463
128	Mustard seed	Oil	292		Derived	Based on #339
129	Poppy seed	Oil	296		Derived	Based on #339
130	Safflower seed	Oil	280		Derived	Based on #339
131	Tallowtree seeds	Oil	305		Derived	Based on #263
132	Tung nuts	Oil	275		Derived	Based on #263
133	Pyrethrum, dried flowers	Others	754		Derived	Based on #558
134	Bambara beans, dry	Pulses	203		Derived	Based on #211
135	Broad beans and horse beans, dry	Pulses	181		Derived	Based on #211
136	Lupins	Pulses	210		Derived	Based on #211
137	Vetches	Pulses	205		Derived	Based on #211
138	Edible roots and tubers with high starch or inulin content, n.e.c., fresh	Roots	149		Derived	Based on #136
139	Yautia	Roots	135		Derived	Based on #136
140	Anise, badian, coriander, cumin, caraway, fennel and juniper berries, raw	Spices	711		Derived	Based on #463
141	Chillies and peppers, dry (Capsicum spp., Pimenta spp.), raw	Spices	689		Derived	Based on #401
142	Cinnamon and cinnamon-tree flowers, raw	Spices	693		Derived	Based on #572
143	Cloves (whole stems), raw	Spices	698		Derived	Based on #572
144	Ginger, raw	Spices	720		Derived	Based on #136
145	Hop cones	Spices	677		Derived	Based on #558
146	Nutmeg, mace, cardamoms, raw	Spices	702		Derived	Based on #572
147	Other stimulant, spice and aromatic crops, n.e.c.	Spices	723		Derived	Based on #463
148	Pepper (Piper spp.), raw	Spices	687		Derived	Based on #401
149	Peppermint, spearmint	Spices	748		Derived	Based on #463
150	Vanilla, raw	Spices	692		Derived	Based on #217
151	Maté leaves	Stimulants	671		Derived	Based on #656
152	Other sugar crops n.e.c.	Sugar crops	161		Derived	Based on #156

153	Artichokes	Vegeta bles	366		Derived	Based on #463
154	Asparagus	Vegeta bles	367		Derived	Based on leafy vegetable
155	Broad beans and horse beans, green	Vegeta bles	420		Derived	Based on #463
156	Cabbages	Vegeta bles	358		Derived	Based on leafy vegetable
157	Cantaloupes and other melons	Vegeta bles	568		Derived	Based on #463
158	Carrots and turnips	Vegeta bles	426		Derived	Based on tuber vegetable
159	Cauliflowers and broccoli	Vegeta bles	393		Derived	Based on #463
160	Chicory roots	Vegeta bles	459		Derived	Based on tuber vegetable
161	Cucumbers and gherkins	Vegeta bles	397		Derived	Based on #463
162	Eggplants (aubergines)	Vegeta bles	399		Derived	Based on #463
163	Green corn (maize)	Vegeta bles	446		Derived	Based on #463
164	Green garlic	Vegeta bles	406		Derived	Based on tuber vegetable
165	Leeks and other alliaceous vegetables	Vegeta bles	407		Derived	Based on leafy vegetable
166	Lettuce and chicory	Vegeta bles	372		Derived	Based on leafy vegetable
167	Mushrooms and truffles	Vegeta bles	449		Derived	Based on leafy vegetable
168	Okra	Vegeta bles	430		Derived	Based on #463
169	Onions and shallots, green	Vegeta bles	402		Derived	Based on leafy vegetable
170	Other beans, green	Vegeta bles	414		Derived	Based on #463
171	Peas, green	Vegeta bles	417		Derived	Based on #463
172	Pumpkins, squash and gourds	Vegeta bles	394		Derived	Based on #463
173	Spinach	Vegeta bles	373		Derived	Based on leafy vegetable
174	String beans	Vegeta bles	423		Derived	Based on leafy vegetable
175	Watermelons	Vegeta bles	567		Derived	Based on #463

References

1. FAO. Definitions. *FAOSTAT database* https://www.fao.org/faostat/en/#definitions (2023).

2. Abrha, B. *et al.* SOWING STRATEGIES FOR BARLEY (*HORDEUM VULGARE* L.) BASED ON MODELLED YIELD RESPONSE TO WATER WITH AQUACROP. *Ex. Agric.* **48**, 252–271 (2012).

3. Bello, Z. A. & Walker, S. Calibration and validation of AquaCrop for pearl millet (Pennisetum glaucum). *Crop Pasture Sci.* **67**, 948 (2016).

4. Ausiku, P. A., Annandale, J. G., Steyn, J. M. & Sanewe, A. J. Crop Model Parameterisation of Three Important Pearl Millet Varieties for Improved Water Use and Yield Estimation. *Plants* **11**, 806 (2022).

5. Yuan, M. *et al.* Assessment of crop growth and water productivity for five C3 species in semi-arid Inner Mongolia. *Agricultural Water Management* **122**, 28–38 (2013).

6. Marsal, J., Girona, J., Casadesus, J., Lopez, G. & Stöckle, C. O. Crop coefficient (K c) for apple: comparison between measurements by a weighing lysimeter and prediction by CropSyst. *Irrig Sci* **31**, 455–463 (2013).

7. Carr, M. K. V. THE WATER RELATIONS AND IRRIGATION REQUIREMENTS OF AVOCADO (*Persea americana* Mill.): A REVIEW. *Ex. Agric.* **49**, 256–278 (2013).

8. CARR, M. K. V. THE WATER RELATIONS AND IRRIGATION REQUIREMENTS OF BANANA (MUSA SPP.). *Experimental Agriculture* **45**, 333–371 (2009).

9. Er-Raki, S. *et al.* Parameterization of the AquaCrop model for simulating table grapes growth and water productivity in an arid region of Mexico. *Agricultural Water Management* **245**, 106585 (2021).

10. Qin, W., Heinen, M., Assinck, F. B. T. & Oenema, O. Exploring optimal fertigation strategies for orange production, using soil–crop modelling. *Agriculture, Ecosystems & Environment* **223**, 31–40 (2016).

11. Amini, A., Karami, F., Sedri, M. H. & Khaledi, V. Determination of water requirement and crop coefficient for strawberry using lysimeter experiment in a semi-arid climate. *H2Open Journal* **5**, 642–655 (2022).

12. de Azevedo, P. V., da Silva, B. B. & da Silva, V. P. R. Water requirements of irrigated mango orchards in northeast Brazil. *Agricultural Water Management* **58**, 241–254 (2003).

13. Carr, M. K. V. THE WATER RELATIONS AND IRRIGATION REQUIREMENTS OF CASHEW (*ANACARDIUM OCCIDENTALE* L.): A REVIEW. *Ex. Agric.* **50**, 24–39 (2014).

14. Azevedo, P. V. de, Sousa, I. F. de, Silva, B. B. da & Silva, V. de P. R. da. Water-use efficiency of dwarf-green coconut (Cocos nucifera L.) orchards in northeast Brazil. *Agricultural Water Management* **84**, 259–264 (2006).

15. CARR, M. K. V. THE WATER RELATIONS AND IRRIGATION REQUIREMENTS OF COCONUT (Cocos nucifera): A REVIEW. *Experimental Agriculture* **47**, 27–51 (2011).

16. Chibarabada, T. P., Modi, A. T. & Mabhaudhi, T. Calibration and evaluation of aquacrop for groundnut (Arachis hypogaea) under water deficit conditions. *Agricultural and Forest Meteorology* **281**, 107850 (2020).

17. Carr, M. K. V. THE WATER RELATIONS AND IRRIGATION REQUIREMENTS OF OIL PALM (*ELAEIS GUINEENSIS*): A REVIEW. *Ex. Agric.* **47**, 629–652 (2011).

18. Paço, T., Paredes, P., Pereira, L., Silvestre, J. & Santos, F. Crop Coefficients and Transpiration of a Super Intensive Arbequina Olive Orchard using the Dual Kc Approach and the Kcb Computation with the Fraction of Ground Cover and Height. *Water* **11**, 383 (2019).

19. Zeleke, K. T., Luckett, D. & Cowley, R. Calibration and Testing of the FAO AquaCrop Model for Canola. *Agronomy Journal* **103**, 1610–1618 (2011).

20. Jahan, M. & Nassiri-Mahallati, M. Modeling the response of sesame (Sesamum indicum L.) growth and development to climate change under deficit irrigation in a semi-arid region. *PLOS Clim* **1**, e0000003 (2022).

21. Carr, M. K. V. THE WATER RELATIONS OF RUBBER (*HEVEA BRASILIENSIS*): A REVIEW. *Ex. Agric.* **48**, 176–193 (2012).

22. Giambelluca, T. W. *et al.* Evapotranspiration of rubber (Hevea brasiliensis) cultivated at two plantation sites in Southeast Asia. *Water Resources Research* **52**, 660–679 (2016).

23. Guang, J. *et al.* Effects of Irrigation Amount and Irrigation Frequency on Flue-Cured Tobacco Evapotranspiration and Water Use Efficiency Based on Three-Year Field Drip-Irrigated Experiments. *Agronomy* **9**, 624 (2019).

24. Espadafor, M. *et al.* Simulation of the Responses of Dry Beans (*Phaseolus vulgaris* L.) to Irrigation. *Transactions of the ASABE* **60**, 1983–1994 (2017).

25. Mubvuma, Michael. T., Ogola, J. B. O. & Mhizha, T. AquaCrop model calibration and validation for chickpea *(Cicer arietinum)* in Southern Africa. *Cogent Food & Agriculture* **7**, 1898135 (2021).

26. Nunes, H. G. G. C. *et al.* Parameterization of the AquaCrop model for cowpea and assessing the impact of sowing dates normally used on yield. *Agricultural Water Management* **252**, 106880 (2021).

27. Kanda, E. K., Senzanje, A. & Mabhaudhi, T. Calibration and validation of the AquaCrop model for full and deficit irrigated cowpea (Vigna unguiculata (L.) Walp). *Physics and Chemistry of the Earth, Parts A/B/C* **124**, 102941 (2021).

28. Paredes, P. & Torres, M. O. Parameterization of AquaCrop model for vining pea biomass and yield predictions and assessing impacts of irrigation strategies considering various sowing dates. *Irrig Sci* **35**, 27–41 (2017).

29. Wellens, J. *et al.* Calibration and validation of the FAO AquaCrop water productivity model for cassava (Manihot esculenta Crantz). *Agricultural Water Management* **263**, 107491 (2022).

30. Montoya, F., Camargo, D., Ortega, J. F., Córcoles, J. I. & Domínguez, A. Evaluation of Aquacrop model for a potato crop under different irrigation conditions. *Agricultural Water Management* **164**, 267–280 (2016).

31. Rankine, D. R. *et al.* Parameterizing the FAO AquaCrop Model for Rainfed and Irrigated Field-Grown Sweet Potato. *Agronomy Journal* **107**, 375–387 (2015).

32. Mabhaudhi, T., Modi, A. T. & Beletse, Y. G. Parameterisation and evaluation of the FAO-AquaCrop model for a South African taro (Colocasia esculenta L. Schott) landrace. *Agricultural and Forest Meteorology* **192–193**, 132–139 (2014).

33. Srivastava, A. K. & Gaiser, T. Simulating biomass accumulation and yield of yam (Dioscorea alata) in the Upper Ouémé Basin (Benin Republic)- I. Compilation of physiological parameters and calibration at the field scale. *Field Crops Research* **116**, 23–29 (2010).

34. Carr, M. K. V. & Lockwood, G. THE WATER RELATIONS AND IRRIGATION REQUIREMENTS OF COCOA (*THEOBROMA CACAO* L.): A REVIEW. *Ex. Agric.* **47**, 653–676 (2011).

35. Kaimuddin *et al.* Water requirement for cocoa (Theobroma cacao L.) plant and the effect of climate factors on the distribution of the cocoa pod borer attacks (Conopomorpha cramerella Snellen) in North Luwu Regency using Cropwat 8.0. *IOP Conf. Ser.: Earth Environ. Sci.* **575**, 012116 (2020).

36. CARR, M. K. V. THE WATER RELATIONS AND IRRIGATION REQUIREMENTS OF COFFEE. *Experimental Agriculture* **37**, 1–36 (2001).

37. Flumignan, D. L., de Faria, R. T. & Prete, C. E. C. Evapotranspiration components and dual crop coefficients of coffee trees during crop production. *Agricultural Water Management* **98**, 791–800 (2011).

38. Pezzopane, J. R. M., Salva, T. de J. G., de Lima, V. B. & Fazuoli, L. C. Agrometeorological parameters for prediction of the maturation period of Arabica coffee cultivars. *Int J Biometeorol* **56**, 843–851 (2012).

39. Batool, D. *et al.* A Hybrid Approach to Tea Crop Yield Prediction Using Simulation Models and Machine Learning. *Plants* **11**, 1925 (2022).

40. Garcia-Vila, M., Morillo-Velarde, R. & Fereres, E. Modeling Sugar Beet Responses to Irrigation with AquaCrop for Optimizing Water Allocation. *Water* **11**, 1918 (2019).

41. Kassing, R., De Schutter, B. & Abraham, E. Optimal Control for Precision Irrigation of a Large-Scale Plantation. *Water Resour. Res.* **56**, (2020).

42. Ćosić, M. *et al.* Predicting biomass and yield of sweet pepper grown with and without plastic film mulching under different water supply and weather conditions. *Agricultural Water Management* **188**, 91–100 (2017).

43. Pérez-Ortolá, M., Daccache, A., Hess, T. M. & Knox, J. W. Simulating impacts of irrigation heterogeneity on onion (Allium cepa L.) yield in a humid climate. *Irrig Sci* **33**, 1–14 (2015).

44. Wellens, J. *et al.* Performance assessment of the FAO AquaCrop model for irrigated cabbage on farmer plots in a semi-arid environment. *Agricultural Water Management* **127**, 40–47 (2013).
